# Perspectives of homeless service providers on their work, their clients, and the healthcare system

**DOI:** 10.1371/journal.pone.0268765

**Published:** 2022-05-26

**Authors:** Cindy Wu Qian, Joshua Hauser

**Affiliations:** Northwestern University Feinberg School of Medicine, Chicago, Illinois, United States of America; PLOS: Public Library of Science, UNITED KINGDOM

## Abstract

**Purpose:**

To describe the perspectives of homeless service providers who work for Chicago organizations that primarily serve persons experiencing homelessness.

**Methods:**

A qualitative, cross-sectional study of Chicago homeless service providers (n = 17) consisting of a semi-structured interview and the Attitudes Toward the Homeless Inventory (ATHI). Interviews were analyzed for themes and patterns using inductive approach.

**Results:**

Four categories of 16 themes describing homeless service providers’ perspectives and perceptions: 1) perspectives on work, 2) perspectives on the general population of those experiencing homelessness at large, 3) perceptions of the population of those experiencing homelessness based on client interactions, and 4) perceptions of hospitals and healthcare.

**Conclusions:**

A richer understanding of the perspectives of homeless service may provide guidance in recruitment and training of workers in this area and offer insight into caring for persons experiencing homelessness in hospital settings.

## Introduction

Sociologists and public health professionals have long recognized structural determinants of homelessness and consequent poor health outcomes among the population of those experiencing homelessness [1]. Prior studies have explored the relationship between homelessness and structural factors including housing market dynamics (rent levels, lack of low-cost housing), poor economic conditions (recessions, high unemployment rates), demographics (race, age cohort), and the presence of safety net programs [2].

While the general population in the United States has increasingly favored structural interventions to combat homelessness [3], recent survey data show that people in the U.S. are less sympathetic toward individuals experiencing homelessness as compared to their counterparts in Canada, where homelessness is equally prevalent [4].

Participating in service work involving interactions with individuals experiencing homelessness seems to have a positive effect on attitudes of participants toward the service recipients. A 2000 study of undergraduate students assigned to work at a homeless shelter compared to control individuals showed increased positive attitudes toward individuals experiencing homelessness [5]. Additionally, a few studies have looked at attitudes of healthcare trainees after intentional clinical exposure to individuals experiencing homelessness during training. After a two-week required homeless health care rotation, primary care residents had a greater belief that homelessness had societal causes and were more willing to interact with individuals experiencing homelessness [6]. Family nurse practitioner students after participating in homeless outreach clinic showed an increase in their beliefs about the social causes of homelessness, an increased belief that more can be done about homelessness, and increased willingness to connect with homeless individuals [7].

What are the perspectives of individuals whose careers involve providing services to persons experiencing homelessness (PEHs)? Few studies have explored the experiences of these individuals, referred to in the literature as homeless service providers (HSPs). A 1991 study examining health care providers’ perceptions of barriers to caring for PEHs found that while providers were supportive of their clients who were experiencing homelessness and rejected that the stigma of homelessness as a deterrent of care, they also believed that characteristics of their clients, namely their lack of motivation and inability to carry out treatment recommendations, could be major barriers to care [8]. A qualitative inductive study of HSPs in a Midwestern city working with homeless noncustodial fathers describes emotional, relational, and systemic factors that affect HSPs’ experiences with their clients, such as a sense of hopelessness, the importance of building rapport and establishing trust with their clients, and the need for a holistic approach to serving this population [9]. Another study exploring perspectives of resident physicians at two large northeastern urban emergency medicine residency programs found that caring for patients experiencing homelessness affects residents emotionally in complex, multifaceted ways, including emotions dominated by frustration and feelings of futility in caring for PEHs [10]. Finally, a study interviewing nurse practitioners on their experiences providing care to PEHs delineated five themes surrounding motivations for their work, challenges to providing care to a unique population with unique needs, values and beliefs of the providers, the evolution of relationship between providers and patients, and lessons learned [11]. Our study will further capture a breadth of attitudes and perceptions that focus on the HSP experience, particularly those employed to serve PEHs.

It is valuable to gain insights into the experiences and perspectives of HSPs, because we know that their attitudes and reactions to their work and their clients are intricately related to the experiences of PEHs. Some studies have examined the perceptions of HSPs from the perspective of PEHs. A 2011 study of mothers experiencing concurrent homelessness and substance use disorders at a Midwestern city shelter showed that these mothers had negative perceptions of service providers, including feeling not understood and feeling judged, desiring more support from service providers, and fear of service providers pertaining to disclosure of information that may cause them to lose their children [12]. A Canadian study interviewed PEHs to explore the nature and meaning of welcomeness and unwelcomeness in health care settings and found that PEHs perceived experiences of unwelcomeness as acts of discrimination against them [13]. Finally, a phenomenological study exploring service encounters from the perspective of women experiencing homelessness described themes of interactions with HSPs that range from dehumanizing to humanizing, dependent on the PEH’s perception of power dynamics and trust with the service provider [14].

Despite the importance of HSPs’ attitudes and perspectives on the experiences of PEHs receiving services, there is little research concerning the perspectives of HSPs who have significant contact with the PEH population. In this study we sought to describe the perspectives of HSPs who work for organizations that primarily target their services toward PEHs in the city of Chicago. According to the Chicago Coalition for the Homeless and the US Census Bureau, approximately 3.2% of the population of Chicago in 2017 were experiencing homelessness [15] [16], and many were likely serviced by organizations in Chicago that provide food, shelter, skills training, healthcare. As of January 2020, there are 91 agencies in Chicago working to end homelessness [17].

In order to more fully understand the lives of HSPs, we explored themes and patterns in their perspectives and attitudes toward their work, their clients, and the healthcare system. Our hope is that this study will help to inform recruitment, retention, and training efforts to focus on relevant and prevalent rewards and challenges that HSPs experience in their work. We also hope to increase awareness for improvement opportunities in the current healthcare system to better serve PEHs.

## Materials and methods

This was a qualitative, cross-sectional study of current HSPs (n = 17) at five Chicago non-profit organizations that predominantly serve PEHs: Chicago Help Initiative; Heartland Alliance Health; Lincoln Park Community Shelter; The Night Ministry; and Pacific Garden Mission. Interviews were conducted face-to-face on-site where the HSPs worked. Each HSP first completed the Attitudes Toward the Homeless Inventory (ATHI) [18] that was administered on a sheet of paper. Each HSP was given as much time as needed to fill out the ATHI by pen without additional commentary or assistance from the author(s) who remained present in the room. After the ATHI responses was collected, the HSPs then immediately completed a half-hour, semi-structured interview conducted by the first author on the same day.

### Ethics approval

This study was approved by the Northwestern University Institutional Review Board (IRB) for Exempt Review (https://www.irb.northwestern.edu/exempt-review/) as data was analyzed anonymously. Written informed consent was obtained for all individual participants, and all participant organizations gave their permission to be identified by name.

### Selection of organizations

We endeavored to have a wide array of perspectives in our sample to mitigate selection bias of our data that may result if we were to only focus on a subset of roles, services, or types or organizations. We deliberately sought out organizations that varied in organizational structure, size, geographic location, affiliation (i.e. faith-based), and services offered. Within each organization, our aim was to diversify on roles and tenure in the field of homeless care. We acknowledge there may still be selection bias even in light of these efforts. And while the diversity allows for better representation of HSP perspectives, it also presents unique challenges to analyzing interview data for common themes across participants and drawing conclusions from the diverse array of backgrounds.

The five organizations were selected to represent perspectives from a diverse sample of service providers to PEHs in Chicago. These included:

- *Chicago Help Initiative*: A volunteer-based organization that provides meals and social services to those experiencing hunger and homelessness
- *Heartland Alliance Health*: A Federally Qualified Health Center that provides comprehensive primary care services to refugees and those experiencing homelessness
- *Lincoln Park Community Services*: A social service agency with two interim housing programs that provide tailored wraparound services to help guests overcome barriers to long term permanent housing
- *The Night Ministry*, *Street Medicine team*: An outreach team that serves individuals experiencing homelessness living outside of shelters by helping with basic needs and providing case management, harm reduction, and medical care
- *Pacific Garden Mission*: A faith-based organization that offers shelter, addiction treatment programs, and medical care for individuals experiencing homelessness

There was no relationship between the researchers and participating organizations. Six organizations were initially identified through internet searches for Chicago non-profit organizations serving individuals experiencing homelessness, with emphasis placed on maximizing diversity of organizational services including shelter, healthcare, food, substance use treatment, and case management. With the exception of Night Ministry, the first author acquired contact information through each organization’s website. HSPs from five of the six organizations were included in the study, with no refusals of participation from any selected HSPs. HSPs from the sixth organization were not identified or interviewed due to difficulty with communication with the organization’s contact person. Participating organizations and each individual HSP understood that the researchers were interested in eliciting HSP perspectives on their work and clients.

### Selection of HSPs

To simplify the logistical efforts for each organization, we asked the contact person from each organization to select HSPs for us to interview. We asked the contact person to select HSPs representing a variety of roles (e.g. front-line, managerial, etc.) and length of time with the organization. The only eligibility criterion was at least part-time employment with the organization. An exception was made for two long-time volunteers at an organization where there was only one employed HSP. Three to 5 HSPs were interviewed at each organization for a total of 17 HSPs. The authors did not consider data saturation in determining sample size but instead focused on researcher feasibility within the study timeline as well as prioritized diversity of HSPs and organizations.

### Attitudes Toward Homelessness Inventory (ATHI)

This is a validated 11-item Likert scale questionnaire containing 4 subscales that assess for Personal Causation (i.e. willingness to affiliate with homeless people), Structural Causation (i.e. belief that homelessness has societal causes), Affiliation (i.e. belief that homelessness is caused by personal characteristics) and Solutions (i.e. belief that homelessness is a solvable problem). Higher scores indicate non-stigmatizing attitudes [18]. (See S1 File for the complete inventory.) Alpha coefficients describing internal consistency within each subscale are 0.72 (Personal Causation), 0.73 (Structural Causation), 0.65 (Affiliation), and 0.60 (Solutions). The alpha coefficient for all 11 statements in the inventory is 0.71. Preliminary validation studies of the ATHI show that subscales collectively accounted for 62–67% of variance in the ATHI [18].

### Development of interview script

The interview questions were developed by the authors based on review of literature about perspectives and attitudes toward PEHs, personal experiences with HSPs and PEHs, and consultation with experts in the field of homeless healthcare.

An initial set of 19 interview questions were piloted with the first two HSPs in our sample. Questions were asked in topical groups in order of priority to the researcher, and the primary purpose of the pilot interviews was to determine the amount of questions that could be answered within a half hour time frame. For both HSPs, we were able to ask all questions in our interview script. For the final interview script, the questions were rearranged in a more logical order after debriefing between the two researchers. HSP feedback was not solicited. Additionally, due to overlap in responses, three questions about HSP perceptions were combined into one and two questions about job entry were combined into one. Finally, two new questions asking about specific examples of interactions with clients were included.

The final interview script consisted of four sections, including: “mapping a typical day,” “motivations for work,” “perceptions,” and “miscellaneous,” totaling 18 questions for an approximately half-hour interview. We asked HSPs to walk us through a typical day in their work lives, and then we focused the majority of our interview on asking about their perceptions of PEHs, their work, and the healthcare system. (See S2 File for the complete interview script).

### Collection of interview data

The first author conducted all of the 17 interviews, and the second author was present for six of these interviews across four different organizations. No other individuals beside the participant and the researcher(s) was present during each interview, and no repeat interviews were conducted. An audio recording was used to collect the interview data, with permission from each participant. Field notes were made during the interview, and no notes contained participant identifiers. Each interview was subsequently transcribed for data analysis, and transcripts were not returned to participants for comment.

### Data analysis

We analyzed narrative data using methods of content analysis and used an inductive approach (grounded theory) [19] to coding (i.e. categories were developed during the coding process) to analyze data for themes and patterns of the interviews. A qualitative data organizer application (Dedoose) was used to assist with coding, and reliability was checked among the two authors. Codes reflecting thematic content of the participants’ answers were developed by the authors based on first few interviews and then these codes were generally applied to subsequent transcripts. However, given the diverse nature of the HSP sample across different organizations and roles, the authors were also intentional about highlighting new and unique patterns and concepts that arose in subsequent transcripts. All transcripts were read by both authors who met to discuss how they coded text. Although no formal mechanism was used to track disagreements about text assigned to different codes, any differences were resolved by discussion. At several points in the process, codes were reviewed and grouped into themes which were further grouped into broader categories.

The final draft of the manuscript for journal submission was shared with participants as courtesy, but no feedback on the findings were solicited.

### Interviewer characteristics and biases

At the time of the study, the first author was a Doctor of Medicine (MD) candidate with prior qualitative data collection, coding, and research experience. The second author is an internist and palliative care physician (MD) who has prior qualitative research experience in areas of palliative medicine. He has over 20 years of experience working with individuals experiencing homelessness, including regular volunteer experience at a suburban homeless shelter (not selected for this study).

The authors were initially interested the research topic out of personal curiosity about the perspectives and attitudes of service workers who regularly interact with individuals experiencing homelessness. Prior to the study, the first author had an encounter with an HSP who shared about her frustration at PEHs and appeared to place blame on some of her PEH clients for the state they were in. This was surprising to the first author, who was under the impression that HSPs, in contrast with the general population and in alignment with many sociologists and public health experts, have significant empathy toward PEHs and attribute the cause of homelessness to systemic failures. The authors sought to explore the generalizability and relevance of these sentiments across a broad diverse array of situations. Thus, many of the questions asked in the interview script initially stemmed from personal interest, and the questions provided a foundation upon which to sort HSP responses into themes and patterns. In the discussion section of this article, we provide further comments on how our own backgrounds and experiences as well as our role as researchers have affected the research.

## Results

### Sample characteristics

We interviewed 17 homeless service providers in a variety of different roles, including 2 volunteers, 3 executive positions, 2 mid-tier director positions, 2 community/outreach roles, 5 healthcare roles (1 Doctor of Medicine (MD), 2 nurse practitioners (NP), 1 registered nurse (RN), 1 medical assistant (MA)), 1 counselor, 1 case manager, and 1 administrative assistant. Of the 17 HSPs, 10 identified as female and 7 identified as male.

Based on their description of their role and day-to-day responsibilities, participants were divided into three categories depending on their interactions with their clients: Direct Service—Non-Health (DSNH), whose role predominantly involves providing non-healthcare services; Direct Service–Health (DSH), whose role predominantly provides healthcare services; and Administrative (AD), who spends 75+% of the time involved in non-direct service work. It is important to note that many in the AD category at the time of the study have spent significant time in direct service roles prior to transitioning to a predominantly administrative role. Table 1 further describes HSP characteristics.

**Table 1:** HSP characteristics.

Role	n	Average years in position (range)	Average years in field (range)	Average hours/week (range)
Direct Service Non-Healthcare Worker (DSNH)	7	4.5 (0.67–17)	8.2 (0.67–17)	32.5 (8–50)
Direct Service Healthcare Worker (DSH)	4	8.1 (1.5–25)	23.3 (3–38)	35.6 (22.5–50)
Administrator (AD)	6	2.7 (1–6)	9.8 (2–20)	43.3 (35–50)

Initially we hypothesized that there would be notable patterns in responses across these three roles (DSNH, DSH, AD given differing levels of exposure and types of interactions with PEHs. While we could not conduct quantitative analyses given sample size limitations, we also did not find any evident differences in ATHI or interview responses between these three groups. This is likely due to the fluidity of roles in our sample, as almost everyone in the AD group had previously worked in more direct client-facing roles prior to their current less client-facing administrative role. Additionally, those in the DSH group that provided healthcare services typically also described non-healthcare-specific tasks such as addressing social needs in their work that at times resembles the roles of those in the DSNH group. Nevertheless, we have disclosed their respective role groups when presenting direct quote examples in our interview results following.

### Attitudes Toward Homelessness Inventory

**Fig 1:**
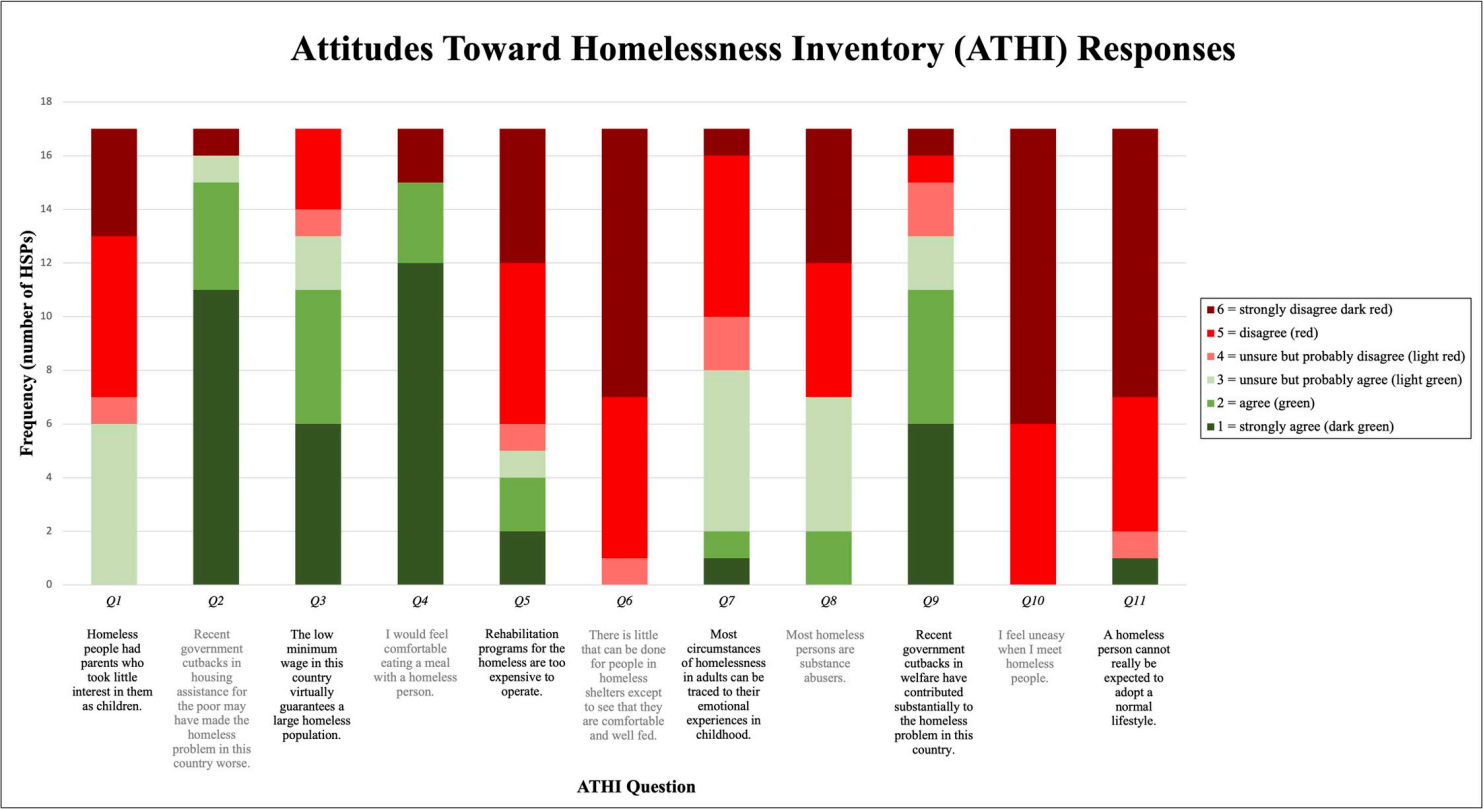
ATHI responses. The distribution of responses across 11 different statements of the ATHI. Red colors indicate disagreement with the statement, and green colors indicate agreement with the statement.

Fig 1 shows the breakdown of HSP responses to the Attitudes Toward Homelessness Inventory (ATHI). The inventory consisted of 11 statements about homelessness to which HSPs rated 1 to 6 in terms of agreement, with 1 = strongly agree, 2 = agree, 3 = unsure but probably agree, 4 = unsure but probably disagree, 5 = disagree, and 6 = strongly disagree. The statements with the highest frequency of “strongly disagree” included “I feel uneasy when I meet homeless people” (Q10), “A homeless person cannot really be expected to adopt a normal lifestyle” (Q11), and “There is little that can be done for people in homeless shelters expect to see that they are comfortable and well fed” (Q6). The statements with the highest frequency of “strongly agree” included “Recent government cutbacks in housing assistance for the poor may have made the homeless problem in this country worse” (Q2) and “I would feel comfortable eating a meal with a homeless person” (Q4).

The score for each ATHI statement was assigned based on HSP response of 1–6. Reverse scoring was implemented for statements 2, 3, 4, and 9 so that high scores would reflect positive, non-stigmatizing attitudes, consistent with high scores for the other statements. Average score and standard deviation across 17 HSPs were calculated for each ATHI statement. The statements were further grouped into 4 belief subscales, and average and cumulative scores for each subscale were calculated. Finally, an overall average and cumulative score for all 11 statements were calculated. Table 2 shows these results, including a range of possible scores.

**Table 2:** Average and cumulative ATHI scores.

Subscale	Statements	Average score	Standard deviation	Range of possible scores
Personal Causation *Belief that homelessness is not caused by personal characteristics*	Q1. Homeless people had parents who took little interest in them as children.	4.47	1.23	1–6
	Q7. Most circumstances of homelessness in adults can be traced to their emotional experiences in childhood.	3.82	1.33	1–6
	Q8. Most homeless persons are substance abusers.	4.35	1.5	1–6
	Subscale average score	4.22	1.36	1–6
	Subscale cumulative score	12.65	3.39	3–18
Structural Causation *Belief that homelessness has societal causes*	Q2. Recent government cutbacks in housing assistance for the poor may have made the homeless problem in this country worse.	5.35	1.27	1–6
	Q3. The low minimum wage in this country virtually guarantees a large homeless population.	4.59	1.5	1–6
	Q9. Recent government cutbacks in welfare have contributed substantially to the homeless problem in this country.	4.59	1.54	1–6
	Subscale average score	4.84	1.46	1–6
	Subscale cumulative score	14.53	3.32	3–18
Affiliation *Willingness to affiliate with homeless people*	Q4. I would feel comfortable eating a meal with a homeless person.	5.24	1.64	1–6
	Q10. I feel uneasy when I meet homeless people.	5.65	0.49	1–6
	Subscale average score	5.44	1.21	1–6
	Subscale score	10.88	1.62	2–12
Solutions *Belief that homelessness is a solvable problem*	Q5. Rehabilitation programs for the homeless are too expensive to operate.	4.29	1.79	1–6
	Q6. There is little that can be done for people in homeless shelters except to see that they are comfortable and well fed.	5.53	0.62	1–6
	Q11. A homeless person cannot really be expected to adopt a normal lifestyle.	5.29	1.26	1–6
	Subscale average score	5.04	1.4	1–6
	Subscale cumulative score	15.12	2.96	3–18
Overall average score		4.83	1.43	1–6
Overall cumulative score		53.18	7.09	11–66

### Perspectives and perceptions: Categories and themes

In our interviews we found 4 categories and 16 themes (Table 3).

**Table 3:** Categories and themes.

**1. Perspectives on work** Themes: • Entry into the field: job versus mission • Motivation: making a difference • Challenges: systemic versus individual behavior **2. Perspectives of general PEH population at large** Themes: • Multiple causes of homelessness • Diversity of PEH population • Housing is most pressing need • Misperceptions of general population • Approaching PEH outside of work **3. Perceptions of PEH based on client interactions** Themes: • Perceptions change over time • “They are just people” • Resilience of PEH • Respect for choices • Ambivalence toward PEH **4. Perceptions of hospitals and healthcare** Themes: • Negative views toward hospitals • Hospitals as entry point • Homeless healthcare organizations

#### Category 1: Perspectives on their work

In terms of backgrounds and how HSPs ended up in the field of homeless service work, 1 HSP described their personal conviction from observations of the PEHs in the community, 1 was influenced by the values of their upbringing, 3 were inspired by past work experiences with other underserved groups, 4 had prior tangential exposures to homeless service work, 2 had personally experienced homelessness, 2 came straight out of training and thought it was a reasonable option in which to apply their training, and 3 were “just looking for a job”.

*Entry into the field*: *job versus mission*. HSPs fell into two general categories with regards to entry into the field of homeless service: “mission driven" (i.e. “doing this work because it has meaning”) versus “job driven” (i.e. “pursuing this job and then finding it meaningful”). In the words of an HSP who pursued their job out of personal conviction,

*My daily commute to school—you would see a lot of them on the streets pan handling or just sleeping in certain bus stops and that kind of tugged at my heart […] what is that I can do to provide help and assistance or to be able to reduce the numbers of homelessness on the streets of Chicago*? *That’s when I started getting involved in any type of homeless outreach*. (HSP 12, DSNH)

On the other hand, others were less intentional initially upon entry into the field of homeless service, but over time they developed a deeper relationship with the field.

*…when I was applying*, *I was just applying for a job because I needed a job at the moment*. *But I stayed because I felt a home*, *I felt like I’m needed*, *like I’m doing something that means something*. (HSP 7, AD)

*Motivation*: *Making a difference*. HSPs felt that their jobs are gratifying and that they are motivated by experiences of witnessing life changes.

*The best part is seeing people get into housing*, *that’s something I’ve always been my favorite part of the job and it never gets old*. *When someone finally gets keys to their own apartment*, *that’s definitely the best part*. *[…] It’s very satisfying knowing you helped make it happen*. (HSP 9, AD)

Some had personally experienced homelessness or difficult situations themselves and found it especially rewarding to be able to “give back.”

*…I truly believe that I’m here and I’m supposed to help others just like somebody helped me*. *They helped me when I was on drugs and tried to get my life together*. *So I’m a firm believer I should be giving that back*. (HSP 15, DSNH)

*Challenges*: *Systemic versus individual behavior*. HSPs described challenges that referenced PEH behavior (e.g. refusal to accept help, participate, comply to rules; drug relapse, mental health barriers), and some comments appear to place blame on PEHs for their circumstances. One HSP commented,

*They are kind of stubborn*. *They do not sometimes listen*. *Let’s say you tell them "ok you have an appointment […]” I give them a list*, *a reminder*, *[…] and if you cannot make an appointment please call*. *The disappointing part is they do not follow through or they do not call after all the work that you did for them*. *[…]After giving them everything*. *"Here’s the appointment" was not typed in 11 font*, *it was typed in 20 font*, *how can you miss that*? *And it’s like there’s no appreciation*. *That’s the disappointing part*. (HSP 17, DSNH)

Another HSP shared about an incident of violence that made it difficult to provide care.

*We did have a client we were seeing on the West side in an encampment*. *He was apparently violent*. *[…] He told another worker that he was going to kill us all*. *And he grabbed a piece of our equipment and ran*, *that was pretty challenging*. *Because now we can’t go back to that encampment and there are other people there who really do need our help*. (HSP 13, DSH)

HSPs also mentioned systemic challenges, particularly the lack of resources and affordable housing.

*Let’s just start with the federal government and its funding and whatever it is these days*, *the lack of state budget—there’s no affordable housing anywhere*, *there’s no subsidized housing*, *HUD is a joke—a neurosurgeon running HUD*? *Like all these policies that are starting to come out*, *they’re downers*. (HSP 6, DSH)

While some comments centered on either the individual PEH or systemic challenges, other comments illustrate challenges that blur the line between individual and systemic.

*A classic example is—people get a mobile housing voucher Section 8 where they can locate any apartment that will take them*, *and they have a limited time to find an apartment*, *and so you say “find an apartment*, *get in the system*, *whether or not you live in it*,*” and they say “no*,*” because many of these are older African American men who want to live on the North Side*. *[…] They want to be in a place where they can live safely*, *and that means most of the time the North Side*. *They’ll say “no I haven’t found an apartment on the North side I like”–[I say] “I don’t care*, *get an apartment*, *get in the system*, *whether or not you ever visit that apartment as long as it’s paid for by Section 8—take it*, *keep it*, *and then transfer out*, *but you have limited time to do this*.*” […] So the argument that’s being made that “you have a limited time*, *here’s the solution to a problem*,*” instead doesn’t result necessarily in people acting on that solution which they could …* (HSP 3, AD)

Here we see that the HSP is frustrated that PEH clients are adamant about finding an apartment on the North Side yet fail to realize that they should prioritize accepting any apartment opening regardless of preference so that they can be in the system at all, as transfers to North Side apartments are significantly easier once they are in the system. However, there is a fundamental systemic issue that underlies the HSP’s frustration toward individual PEH behavior—that there is an insufficient amount of affordable housing options available in safer neighborhoods. Thus, we see how systemic barriers cause problematic individual reactions that are difficult for HSPs to navigate.

#### Category 2: Perspectives on general PEH population at large

*Multiple causes of homelessness*. HSPs acknowledged that there are many different factors that could lead a person to experience homelessness, and each individual may come from different situation. Common factors stated by HSPs include mental illness and substance use.

*There’s a huge variety of people—they bring a huge wealth of talent*, *a huge wealth of life experience*, *recurrent themes again are addiction and mental illness in the broadest possible definition*. *[…] I remember talking to a guy who leaned over one time and said–“I don’t tell everybody this but my name is actually Han Solo*.*” I’ve got a guy named Vern Superfoods*, *can’t be his real name*. *So that’s a clear mental illness*, *paranoia*, *delusions that people can bring with them…* (HSP 3, AD)

This HSP additionally noted,

*… a huge proportion of people who come to us have a very limited or small families*. *One of the things we rely on to get us out of trouble is often family and if you don’t have family that often isn’t there*. *So if I were to lose my job*, *my sister wouldn’t turn me out*, *my brothers wouldn’t turn me out*, *[…] if I didn’t have family around me*, *my resources become much less*. *So resources have to be thought about in terms of social connection and family connection and not just income*. (HSP 3, AD)

Other HSPs cited systemic and political roots as causes of homelessness.

*I can give you the standard answer—it’s multifactorial*, *nobody has just one cause of homelessness*. *But why are people homeless*? *It’s because our economy is horrible and we treat poor people bad and we suppress them*. *We just need a revolution—people are homeless because there’s no low-income housing*, *because the state hasn’t had budget*, *because Donald Trump exists*, *because White House* . . . *and it’s getting worse*, *but it’ll probably get worse before it gets better*. (HSP 6, DSH)

*Diversity of PEH population*. HSPs noted that PEH population is more than the “single man on the streets begging for money” that the public typically encounters, including children, families, working poor, refugees, and “couch surfers”.

*It’s a wildly diverse group of people*, *which I wouldn’t have had visibility on from my previous view which was mostly street homelessness and people panhandling*, *the sort of "visible face" of things—there are a lot of families*, *a lot of young folks…I think that’s the biggest change in impressions—from the single man usually on the street visibly poor asking for money or very visibly mentally ill to a good portion of our folks who just don’t have access to healthcare and mental health or substance abuse—or other drivers are not their issue at all*, *they’re just very poor…*(HSP 4, AD)

Some HSPs distinguished a subgroup of PEH commonly referred to as “rough sleepers” who avoid shelters and instead spend their nights on the streets or in encampments.

*There’s this impression that people want to be on the streets*. *I think that’s exceedingly rare*. *I’ve not heard it yet*, *but I do hear people choosing not to be in shelters with a lot of frequency*. *Couples often can’t be in shelters*, *people with pets can’t be in shelters*, *they’re pretty dangerous places to be*. *If you add a little paranoia into the symptoms of what someone’s living with*, *being in a shelter is a very difficult place to be*. *So people often choose streets vs shelters*, *but that doesn’t mean they want to be on the streets necessarily*. (HSP 4, AD)

*Housing is most pressing need*. Among the many needs of the PEH population, housing was believed to be the most pressing.

*…there are lots of needs*, *but being on the streets makes everything worse*, *and getting off the streets makes everything better—you can store proof*, *you can store your personal papers*, *you’re safe*, *you’re not constantly moving*, *you’re not constantly threatened—it’s such a huge difference*. (HSP 3, AD)

*Misperceptions of general population*. According to HSPs, the general population’s most problematic misperception is that everyone in the PEH population is “on drugs or alcohol” and viewed as “lazy.” HSPs urged the public to take time to see who these people really are.

*A lot of people think it’s their problem*, *they picked it*, *they’re lazy—that’s not necessarily the truth*. *There’s a lot of things that go deeper*. *The root of the situation is much worse than what they think*, *it’s not just all surface*. *You have to dig deeper to see what’s really causing their problem—mental state*, *et cetera et cetera…*(HSP 2, DSNH)

Other HSPs said that another problematic misperception is that it is easy to get out of homelessness.

*People tend to believe that it’s just easy to one day say "you know what*, *I’m homeless right now*, *I’m going to change that*, *I’m going to get a job" because you hear tons of people say "hey go get a job" or "this is by choice" "get yourself out of the streets yourself" things of that nature*. *People tend to disregard the fact that it does take an emotional toll on you*, *just like when you lose a parent or when you lose a child or something happens in your life that gives you some type of trauma*. *I believe that every person in this world will at some point in time face some level of depression*, *will face some level of emotional crisis or have an emotional breakdown*, *and we all become vulnerable*. *Unfortunately some people aren’t mentally or emotionally strong enough to get themselves out of that hole*, *they need a support system*. *But if you’re consistently reminded that you’re not valuable*, *that you’re in a position you’re in out of choice*, *you tend to fall deeper into that hole*. *[…] So it’s not as easy as it seems to try to get themselves out of the streets*, *out of the funk that they’re in*. (HSP 12, DSNH)

*Approaching PEH outside of work*. Almost every HSP said that when approaching PEH on the streets asking for monetary help outside of their work, they would engage the PEH in some manner.

*Depends on circumstances and where I’m at*, *where I’m going*, *what time I have available*. *If I’m driving I don’t generally like do anything but smile and make eye contact*. *On the street every once in a while I’ll sit down and just ask someone what’s going on*, *what’s up*. *Every once in a while I’ll give money*. *I don’t know that there’s any rhyme or reason*, *it’s generally indicated by the level of time I have available*, *but I will always smile and make eye contact*, *say hello*. *Always always always*. (HSP 11)

Many would also refer PEHs to services provided by the organization they work for.

*We have cards that I give out that direct them here*. *I will buy someone something to eat*, *but I won’t give them money*. *Because I feel like I’ve been manipulated in that way before*, *so now if you’re hungry I will feed you*, *I will point you in a direction where you can get housing or a bus card*, *but I won’t just give you money*. (HSP 7, AD)

HSPs acknowledged that PEHs encountered on the streets often use money for drugs or alcohol. Most thus choose not to give cash.

*I’m very sad that in this country—and that really bothers me—that people are out on our streets for one reason or the other*. *I do not on the streets however*, *give them money*. *[…] I’ve had several of them say to me*, *“don’t give me money*, *I’ll use it for the wrong thing*.*” It makes me sad*. (HSP 1, DSNH)

In light of this, some HSPs said that they would still give cash to PEHs they encounter out of respect of choice. One HSP reasoned from her own personal experience.

*I usually give it to them if I have*. *A friend of mine said "well no*, *don’t give them because they just gonna’ buy drugs with it" and I always say "well I don’t know what they’re gonna do*, *I don’t know their situation but I used to panhandle too*. *And somebody gave to me*.*"* (HSP 15, DSNH)

#### Category 3: Perceptions of PEH based on client interactions

*Perceptions change over time*. HSPs acknowledge that as they have been exposed to the field of homeless care through interactions with their clients, their perceptions have changed over time. Some elaborated on the importance of the “human connection”:

*I used to be very weary of going up to a homeless person and try to stay away*, *and now I think they just want you to say hello*, *because a lot of the people that are here are not ones you necessarily see on the streets*. *These people either live far away or they stay far away*, *they’re not the people you see in our neighborhood that are just sitting out begging for money*. *I used to be afraid of homeless people*, *kind of like sheering away*. *Now whenever I see somebody that looks familiar I’m like running up to them to say hello*, *I approach them in the streets instead of running away*. (HSP 2, DSNH)

Other HSPs shared that as they have worked with this population they have found that “it is not always what it seems.”

*I came here thinking that people who use drugs and are addicted to drugs are a certain way*, *and I don’t think that anymore*. *Like we have one person who was started on heroin when he was 9*, *by his mom*. *So when you know that back story—and what happened to his mom*? *Why was she so damaged that she couldn’t see that that wasn’t the right thing to do*? *I feel like that really changes you when you see what people have gone through*. (HSP 8, DSH)

HSPs came to empathize and relate to the circumstances of their clients, acknowledging that they themselves are only “a paycheck away” from experiencing homelessness.

*…even though I may not be homeless today*, *but there are many people out there who are literally one paycheck away from being homeless*. *And I know that if I was in that position I would desire some type of grace or compassion to be shown toward me*. *So I try to have that perception of just being very compassionate no matter who it is*, *what it is*, *or what you may think instantaneously of what it could be*. (HSP 12, DSNH)

*“They are just people”*. This expression came up during several interviews, as HSPs described their clients as no different than any other individual in society.

*Everybody has their own story about what happened with them*, *so my perception is just that this is just a human being who had different things happen in their life and this is where they are now*. (HSP 14, AD)

Another HSP said,

*I’ll just say that people who are experiencing homelessness are just people who have been denied housing or haven’t had access to housing for a multitude of reasons*, *but when it comes down to it—homelessness is just a housing status and doesn’t really define anyone in any other way*. (HSP 10, DSNH)

*Resilience of PEH*. HSPs applauded PEHs for their talent, intelligence, and strength amidst the circumstances they face.

*…they’re very very intelligent human beings*. *I think that people have a myth or say things like they’re lazy—they’re just not*. *[…] They just need to be connected to somewhere where they can utilize resources and the staff is highly trained and is compassionate and have empathy to work with the individuals that is just having just a little itty bitty challenges going through in their life*. *Just because they’re not housed*, *does not mean they can’t be successful*. (HSP 5, DSNH)

Another HSP shared,

*There’s one person in particular I have met and know who has just been through a lot of trauma in their life and really don’t have the social support I have and probably too often take for granted*, *and just seeing this person come through what they have come through and still are able to maintain a positive outlook and persist in achieving their goals and want to give back to the community and have gratitude—I’m thinking of a specific person but at the same time I’m realizing that that happens often*. (HSP 10, DSNH)

*Respect for choices*. HSPs described the approaching clients as dignified individuals whose decisions and choices should be respected.

*We have people that use drugs that still want to use drugs*, *and we want to support them*, *that’s their choice*. *If they want to continue using heroin*, *how can we ensure that you’re not going to get HIV or hepatitis and respect them that they are valued and they can make the choices that they deserve to make*? *I just think that it’s challenging on a daily basis to meet people where they are and to convince them that you are not there to do more harm*, *that you are there to respect them and to treat them with kindness and compassion*. (HSP 8, DSH)

*Ambivalence towards PEH*. In responses where the HSP described PEHs in a negative light (e.g. angry, demanding, no appreciation, stubborn), the statements were immediately followed by a rationale.

… *many times they’re demanding and that’s okay […] I feel that this is a secure place for them and this is someplace that they have some control*. *New volunteers have to realize that because they’ll find them offensive and demanding and that they do seem a little abrupt at times*. *[…] …many of them do not open up to you until they’re familiar with you and make sure that*, *you know*, *you just aren’t a fly-by-night*, *that you’re trying to know them and even after that time*, *they’re very hesitant at many times*. *You have to get through that*. (HSP 1, DSNH)

One HSP described a silver lining in moments of frustration.

*… a lot of them don’t try to get out of the situation they just are so comfortable with… some of them try to go to education programs we offer them and try to better themselves but some of them are just comfortable with just the status quo and coming back and coming back—makes me feel that some of my work is not really going that far*, *but on the other hand it makes me feel like at least I’m making them feel good for what it’s worth*. (HSP 2, DSNH)

#### Category 4: Perceptions of hospitals and healthcare

*Negative views toward hospitals*. HSPs’ perceptions were frequently negative, recounting anecdotes of poor treatment of PEH in emergency department and hospital settings.

*We know of more than one person who has gone to a place and been kicked out*. *We actually brought somebody to a place once because we were told that’s what we could do*, *and when they saw that they were homeless and dirty*, *they took them from the front of the building where we dropped them off and they walked them through the building and let them out through the back door so they immediately got them out of the building…* (HSP 14, AD)

Another HSP described attitudes toward PEH in the hospital setting as such:

*It’s often easy to distance from people who are homeless and come through the door and like "oh there’s that person again" or "they’re on drugs"—something that pushes them away*. *So hospitals are super unsatisfying for homeless people*, *they don’t have great resources to connect people to*, *and building relationships with [homeless service organizations] has been also pretty tricky*. (HSP 4, AD)

Of the positive perspectives noted in HSP interviews, many included being able to obtain insurance.

*I think now it’s funny there are a lot of people wanting to help the homeless get healthcare*. *Free*. *The healthcare*, *like the Obamacare*, *all the Medicaid plans*, *they’re really good plans*. *People get really good care*, *they don’t have any co-pays*. *I always tease them I say "boy I wish I had your insurance*.*" […] If you can get them signed up*, *they have good healthcare*. (HSP 16, DSH)

A few HSPs also mentioned a Chicago Emergency Department housing initiative based at University of Illinois, Chicago (UIC) [20].

*I think that it’s been cool to see some of the hospitals stepping up*, *even though it’s strictly out of fiscal solutions or wanting to save money on their end*, *but I think it’s pretty awesome that they’re creating housing for some of the most frequent ER visitors that are housing insecure*. *So I really like that because it’s creating a real solution*. (HSP 11, AD)

*Hospitals as entry point*. HSPs believed hospitals could partner more with organizations and refer PEHs for follow-up on health or non-health related needs.

*Every once in a while I’ll get a call from a hospital*, *somebody from the hospital looking to get help for somebody and it’s just clear that they’re not very familiar with homeless services or homeless systems*. *[…] Hospitals are one of the areas where people can go to get referred to a shelter*. *Some people who work in hospitals don’t realize that’s the case*. (HSP 9, AD)

According to another HSP, hospitals could also help by recording homelessness to provide accurate data of homeless healthcare needs.

*Let’s teach everybody who puts a diagnosis in to use that homeless diagnosis code*, *because if you can prove to the insurance companies how many people are actually experiencing homelessness*, *there may be more money from the managed care organizations for housing and to keep people out of the ER and hospitals*, *it’s going to be cheaper to house them than it is to pay for their hospital bills*. *[…] But people need to code homelessness as a diagnosis*. *It isn’t one [right now]*, *and they need to know it’s there*. (HSP 6, DSH)

One HSP talked about the nuances of identifying homelessness in the hospital.

*They can record homelessness better […] One great unknown is actually how many homeless people there are […] HUD defines homelessness as streets and shelters*. *HRSA is a broader definition including all instability—renting by the week*, *renting by the month*, *sleeping with family*, *couch surfing*, *etc*. *So hospitals could record that in a more sensitive way*. *If you ask someone if you’re homeless*, *it’s incomplete by definition […] You have to have better ways of generating that data*, *understanding what homelessness is*. (HSP 4, AD)

*Homeless healthcare organizations*. Some HSPs noted that federally qualified health centers (FQHCs) provide great services to PEHs but have limited mental health services and need more funding.

*We don’t find it particularly challenging to find people healthcare*. *We have a good relationship with a health clinic […] we actually have a nurse practitioner come every week to see our guests*, *and she can facilitate appointment referrals*. *So we don’t have as much of a challenge getting primary health care*. *It can be more of a delay trying to get people an appointment with psychiatric services*, *mental health care*. (HSP 9, AD)

## Discussion

This study has described the perspectives of a diverse group of HSPs and documented their work experiences and attitudes toward the field and their clients. In both the ATHI and our open-ended interviews we found a range of opinions and experiences that overall suggest that HSPs have compassionate attitudes toward their clients and the population of those experiencing homelessness while expressing frustration at systemic issues and individual biases against this population. Below we summarize major findings and consider implications of our results.

Quantitative data from the ATHI showed that on average HSPs score highest on willingness to affiliate with homeless people, followed by beliefs that homelessness is a solvable problem and that it has societal causes. HSPs feel least strongly that homelessness is not caused by personal characteristics. These results complement our findings from interview responses in which HSPs describe empathic attitudes toward their clients experiencing homelessness while acknowledging the role of individual factors (mental illness, substance use, etc.) in entry into homelessness.

For comparison, there are three studies we are aware of that have used the ATHI to assess change in attitudes. An evaluation of primary care residents showed average total scores of 45.4 and 51.7 pre- and post- homeless healthcare-focused curriculum [6] while another study with internal medicine residents showed average total scores of 46.0 and 53.0 pre- and post- intervention [21]. A study of nurse practitioner students showed average scores per ATHI statement were 3.88 and 4.38 pre- and post- participation at a homeless outreach clinic [7]. In comparison, the average total score for HSPs in this study was 53.2 and the average score per ATHI was 4.83, which are comparable to that of the post-exposure scores in these studies. These high baseline ATHI scores are expected of our sample of HSPs whose interview responses show overall positive attitudes toward their clients and the general population of those experiencing homelessness.

In our qualitative analysis of open-ended interviews, we described the views of homeless service providers (HSPs) in four categories that consisted of 16 themes in regard to their perspectives on their work, their clients, and homeless healthcare. These categories developed from their interviews included (1) perspectives on work, (2) perspectives of general PEH population at large, (3) perceptions of PEH based on client interactions, and (4) perceptions of hospitals and healthcare.

### Perspectives on work

With regard to entry into the field, HSPs generally came from two paths: one in which they were intentionally seeking a meaningful career, and the other in which they were merely looking for a job and found themselves working with this population. Regardless of entry method, almost all HSPs found satisfaction and meaning in being able to make a difference in the work that they do. In describing challenges, HSPs expressed frustration at both systemic factors (e.g. lack of affordable housing, political agendas) and also acknowledged issues of mental health, substance abuse, and unemployment. This is consistent with Seiler & Moss’s findings in their interviews with nurse practitioners, who described making a difference as a motivator for their work and also acknowledged the complexity of providing care to this population due to mental illness and substance use [11]. HSPs recognized the challenges and frustrations that come with their work, yet their motivations stemmed from a genuine desire to help individuals experiencing homelessness and are fed by their rich experiences and encounters with PEHs that make their work ultimately redeeming. We see a similar mix of sometimes conflicting emotions seen among emergency medicine residents in Doran et al’s study where residents are frustrated by the limits of their ability to impact PEHs and yet find the work very rewarding [10]. The findings in our study suggest that for most HSPs, the meaning and inspiration of their work are key reasons for their continued connection to the field.

### Perspectives of general PEH population at large

In describing the general PEH population, HSPs urged people to consider individuals experiencing homelessness in a more nuanced way than the stereotypical image of “street homelessness and people panhandling” (HSP 4, AD). They described that these individuals come from diverse backgrounds and circumstances, including those who work full time and yet do not make enough income to afford housing, those who find themselves facing multiple economic and social stressors (e.g. divorce, family death, fire) that led to housing insecurity and homelessness. A study of causes of homelessness among older individuals newly experiencing homelessness found similar patterns across urban areas in three countries- the US, England, and Australia. The study found that participants became homeless through a combination of personal problems (e.g. death of a close relative or relationship breakdown), welfare policy gaps, and service delivery deficiencies [22].

When it came to approaching PEHs outside of work, HSPs made a distinction between this setting and the PEHs with whom they worked directly. Some HSPs worried that some of the individuals who ask for money on the streets do so as part of substance use disorder. One HSP said that someone with a sign on the street had admitted to her that the message and story on their sign was fabricated. Almost half of the HSPs said that they do not to give money to PEHs, while the rest said that they may give money though not every time. Almost all HSPs said they would typically engage PEHs outside of work, at least saying hello and acknowledging them each time. These findings seem to imply that HSPs apply their positive attitudes toward their clients to their response to PEHs on the streets asking for money, but HSPs vary in whether they give money. From the perspective of PEHs who “panhandle” in public spaces, PEHs describe a range of interactions with passersby, including being subject to acts of violence and receiving kindness and support while panhandling. They call for greater empathy, saying that “even a smile helps” [23].

### Perceptions of PEH based on client interactions

HSPs noted that their own perceptions have evolved over time as they have worked in this field, as they reflect on stories of PEHs as victims of unfortunate life circumstances and factors outside their control. There was a common pattern of acknowledgement that PEHs are “just people” and that as HSPs have gotten to know these individuals, they have been moved by stories of incredible resilience amidst struggle. HSPs also described PEH attitude and behavior concerns (e.g. stubbornness, violence), suggesting that these were reasonable given difficult circumstances and emphasizing empathy and respect of choice. HSPs from Rogers & Rogers’s study similarly call for compassion and increased sensitivity by providers, describing something akin to “meeting clients where they were” [9]. The findings in our study show a resistance to stereotypes as HSPs recognize the diverse behaviors and experiences of their clients. This has implications for how HSPs are recruited and introduced to the work, such as emphasizing the opportunity to learn from the unique perspectives and experiences of PEHs.

### Perceptions of hospitals and healthcare

Many HSPs expressed frustration and disappointment in hospital management of PEHs. They recounted situations of inappropriate treatment and recommended inclusion and bias training for staff. Experiences of PEHs in the healthcare system are extensively explored in empirical literature. PEHs have described being dehumanized and treated with disrespect as well as feeling invisible, labeled, and stigmatized by healthcare providers who lack compassion for them [24–26].

HSPs also offered practical suggestions including increasing identification of homelessness (as hospitals are often entry points into help for PEHs) and adequate referral to organizations with expertise in assisting these individuals. Though HSPs noted that hospitals have at times failed to treat PEHs appropriately, they were pleased with homeless healthcare organizations that properly serve PEHs respectfully and competently and also acknowledged the efforts of hospitals in Chicago to house emergency room utilizers who experience homelessness [20]. Finally, HSPs understood that while medical care is important for someone experiencing homelessness, greater priority should be placed on housing PEHs as this intervention would significantly improve their overall physical health. This is an area that others have identified and that might have the most concrete implications from our study: there continue to be studies around identifying “housing first” as a remedy for homelessness [27]. The perceptions of the HSPs in this study also suggest that more specific policies and procedures from hospital emergency departments may be a valuable set of interventions to consider.

### Researcher reflections

It is an important feature of qualitative research that it is impossible to completely remove the researcher from the research [28]. This is a challenge, but can also add to the richness of this method. The authors acknowledge that our position as researchers may have influenced participant responses. For example, it is possible that HSPs felt the need to defend the work they do and the clients they work with because of their notions of common misperceptions about PEHs in the general population. This may have been evident in the “ambivalence toward PEH” theme, in which HSPs described PEHs in a negative light but quickly followed with a rationale, as if to defend their negative sentiments and their PEH clients.

As discussed in the methods section, the authors recognize that our backgrounds and experiences may have informed the structuring of the study. It is also important to acknowledge that similarly our experiences may have influenced the overall interpretation of the data. The two authors represent Asian-American and White-American racial backgrounds as well as identities in dominant categories (e.g. mental and physical ability, sexual orientation, religion, socioeconomic status, education). We have never personally experienced housing insecurity or homelessness and do not know of any close friends or family members who have had such experiences. Additionally, we also bring perspectives as physician and medical student with experiences working with PEHs in medical and hospital contexts. These backgrounds and perspectives may have helped us to ask particular questions and analyze responses, but they may have also created blind spots that hindered our ability to extract insights from the data. It is difficult to exactly articulate our influence over the outcome of the study, so we share these thoughts and encourage readers to carefully examine the data and conclusions.

### Limitations of the study

#### Sample

Due to the qualitative design of the study, this study does not provide a representative sample of perspectives of HSPs, but rather an in-depth consideration of the perspectives of a small group of workers. HSPs interviewed were selected by each organization’s contact person, some of whom intentionally referred us to longtime or seasoned employees, suggesting a possible bias of perspectives in our sample. A more open recruitment strategy would have mitigated some of the selection bias present this study.

#### Timing of administering ATHI

For each of our HSP participants, we first administered the ATHI and then immediately followed with the interview component. Thus, it is possible that the HSPs may have been primed by the ATHI statements when giving open-ended responses to the interview questions, thus influencing the interview data compared to a scenario where HSPs complete the ATHI after the interview. The advantage of completing the ATHI first is that it allows HSPs to begin to contemplate their opinions to the statements that can be further elaborated during the interview. For example, the ATHI poses statements pertaining to personal causation versus structural causation of homelessness, and the interview sought to elicit perspectives around causes of homelessness and thus nicely built off of the ATHI.

#### Interview length

Half an hour may not have been enough time to elicit perspectives of HSPs fully. Our pilot interviews suggested that the questions could be completed within the time frame, but some HSPs elaborated on answers at greater lengths and were unable to complete all 18 questions though all HSPs gave responses to questions within each of the four main sections of the interview script. As a result, our data collection resulted in fewer than 17 unique responses to each of the 18 questions asked, though all relevant topics were discussed with each HSP.

#### Organizational patterns

HSPs who work for the same organization tended to respond in similar patterns and themes. For example, HSPs from a faith-based organization had responses that incorporated faith as part of their motivation for working with this population. HSPs from a healthcare organization were able to discuss homeless healthcare in more depth.

## Conclusions

Despite these limitations, this study provides a rich description of HSP experiences and their attitudes toward their work, PEHs, and homeless healthcare, suggesting overall positive attitudes toward the population of those experiencing homelessness while having mixed attitudes regarding healthcare for these individuals. This study provides a unique set of voices from an important group of stakeholders in the homeless service industry. As various sectors, including healthcare, assess and address the needs of individuals experiencing homelessness, it will be helpful to gain the perspective of those whose careers entail serving this population. Specifically, the insights and advice offered by HSPs regarding healthcare for PEHs may provide initial ideas for medical training curricula and hospital partnership with organizations to serve this population. At our affiliated medical school, for example, this study helped to lay the foundation for a two-week clinical rotation in homeless healthcare at one of our participating organizations.

Additionally, a richer understanding of HSPs’ experiences may serve as guidance in recruitment and retention of workers and volunteers in this area. Literature has shown that about one-third of those working in human service sectors such as homeless care experience burnout [29, 30], which may be a potential explanation for the high turnover rate seen in these jobs. While burnout is significant in the homelessness sector, there is evidence to suggest that organizations can play a role in protecting workers against burnout. One study found that availability of perceived relevant training was positively associated with service provider work engagement and negatively associated with burnout [31]. Another study introduced the “Florence Nightingale effect” that higher perceived client suffering was correlated with higher job satisfaction and lower burnout among staff, and this relationship was mediated by greater identification with the organization [32].

In our interview data, we saw that HSPs expressed frustration toward systemic challenges as well as difficult individual PEH behaviors, and both can be reasonably expected to be associated with burnout. However, none of these themes were explicitly linked to any concerns about burnout or job dissatisfaction in the interview responses. Instead, our study portrayed HSP motivations to serve the PEH population despite challenges in their work, including a strong desire to help and success stories that fuel HSPs in the midst of frustration and potentially shield from burnout. Further studies exploring perspectives of former HSPs who have left their positions and the human service sector altogether may provide insight into understanding retention and turnover as well.

## Supporting information

S1 File Attitudes Toward Homelessness Inventory (ATHI). (PDF)

S2 File Interview script. (PDF)
