# Supplementary material for: Perspectives of homeless service providers on their work, their clients, and the healthcare system
Source: PLoS One. 2022 May 26;17(5):e0268765. doi: 10.1371/journal.pone.0268765 (PMC9135197; doi:10.1371/journal.pone.0268765)
Supplement: S1 File — (PDF) [file pone.0268765.s001.pdf]

S1 File. Attitudes Toward Homelessness Inventory (ATHI).

Attitudes Toward Homelessness Inventory (ATHI)

On the questionnaire, please circle the number that most closely represents your view

1= strongly agree
2=agree
3=unsure but probably agree
4=unsure but probably disagree
5=disagree
6=strongly disagree

1. Homeless people had parents who took little interest in them as children	1	2	3	4	5	6
2. Recent government cutbacks in housing assistance for the poor may have made the homeless problem in this country worse	1	2	3	4	5	6
3. The low minimum wage in this country virtually guarantees a large homeless population	1	2	3	4	5	6
4. I would feel comfortable eating a meal with a homeless person	1	2	3	4	5	6
5. Rehabilitation programs for the homeless are too expensive to operate	1	2	3	4	5	6
6. There is little that can be done for people in homeless shelters except to see that they are comfortable and well fed	1	2	3	4	5	6
7. Most circumstances of homelessness in adults can be traced to their emotional experiences in childhood	1	2	3	4	5	6
8. Most homeless persons are substance abusers	1	2	3	4	5	6
9. Recent government cutbacks in welfare have contributed substantially to the homeless problem in this country	1	2	3	4	5	6
10. I feel uneasy when I meet homeless people	1	2	3	4	5	6
11. A homeless person cannot really be expected to adopt a normal lifestyle	1	2	3	4	5	6
