# Supplementary material for: Perspectives of homeless service providers on their work, their clients, and the healthcare system
Source: PLoS One. 2022 May 26;17(5):e0268765. doi: 10.1371/journal.pone.0268765 (PMC9135197; doi:10.1371/journal.pone.0268765)
Supplement: S2 File — (PDF) [file pone.0268765.s002.pdf]

S2 File. Interview Script.

Mapping a typical day

Now, let's start with general descriptions of what your work looks like. The first question is:

1. Walk me through a typical day in your work life (e.g. administrative, direct contact, etc.). How do you divide up your time?
2. What kind of interactions do you have with your clients who experience homelessness? (i.e. What specific activities and/or interventions?)

Motivations for work

3. How did you get into working with persons experiencing homelessness? In this job specifically?
4. What is particularly rewarding or fulfilling about the work that you do?
5. Can you recount a specific example of an interaction with a “client” you recall as particularly rewarding or fulfilling?
6. What are some challenges that you face in this work and your interactions with persons experiencing homelessness?
7. Can you recount a specific example of an interaction with a “client” you recall as particularly challenging or difficult?
8. What is something that you have learned about in working with this population?
9. What continues to motivate you to work with this population?

Perceptions

Now we're going to ask you some questions about your perceptions of your clients and the population of those experiencing homelessness in general.

10. Based on your experiences, what are your current perceptions of people experiencing homelessness?
11. In your opinion, why are people on the streets? (i.e. why are people in the circumstances they are in, with housing instability?)
12. How do you approach people who panhandle or ask you for money on the streets outside of your work?
13. In your opinion, what is the general population's most problematic misperception of people experiencing homelessness?
14. In your opinion, what are the most pressing needs of the population of those experiencing homelessness right now?
15. In your opinion, what do you wish other people who do not work with this population would know about the work you do?

Miscellaneous

I just have several more miscellaneous questions for you before we wrap up our interview.

16. What are your perceptions of homeless healthcare (i.e. what are your attitudes toward hospitals and hospital systems and how they have provided healthcare to the homeless?)
17. How do you think hospitals can better assist your work in helping this population (aside from what they are already doing e.g. providing shuttle rides, etc.)?
18. Finally, is there anything else that is important to know about your work with individuals experiencing homelessness?
